# Molecular Mechanism of Tetramethylpyrazine Ameliorating Neuroexcitotoxicity through Activating the PKA/CREB Signaling Pathway

**DOI:** 10.1155/2022/2812839

**Published:** 2022-01-20

**Authors:** Hongxuan Tong, Kaili Wang, Xiting Wang, Tao Lu

**Affiliations:** ^1^Institute of Basic Theory of Chinese Medicine, Chinese Academy of Chinese Medical Sciences, Beijing 100700, China; ^2^School of Life Sciences, Beijing University of Chinese Medicine, China

## Abstract

**Background:**

Excitotoxicity plays a key role in nervous system disease and can trigger a critical cascade of reaction which affects cell viability and promotes neuronal death. Tetramethylpyrazine (TMP) reveals its effect in the treatment of neurovascular diseases by antiapoptosis. Recently, there were several studies that demonstrated that the PKA/CREB signaling pathway played a role in neural disease because of excitotoxicity, such as stroke, AD, and Parkinson's disease. In this study, we wanted to focus on the protective effect of tetramethylpyrazine against excitotoxicity through the PKA/CREB signaling pathway.

**Methods:**

In order to verify whether tetramethylpyrazine can attenuate excitotoxicity through the PKA/CREB signaling pathway, we first used molecular docking technology to predict the combinational strength and mode of tetramethylpyrazine with the proteins in the PKA/CREB signaling pathway. Then, we determined the optimal concentration and time according to the model effect of glutamate (Glu) with different concentration gradients and action times in PC12 cells. After the determination of concentration and time of glutamate in the previous step as the model way, tetramethylpyrazine was added to determine its influence on the cell viability under different doses and times. The TUNEL assay and flow cytometry were used to detect apoptosis. RT-PCR was used to detect the expression of Bcl-2, Bax, PKA, and 5CREB genes, and Western blot was used to detect the expression of these factors.

**Result:**

Tetramethylpyrazine had a good docking score (-5.312) with PKA and had a moderately docking score (-3.838) with CREB. The CCK-8 cell activity assay showed that the activity of PC12 cells decreased gradually with the increase in glutamate concentration and time, and PC12 cells were treated with 10 mM/L glutamate (the half of the inhibitory concentration (IC50)) for 12 hours. Then, the cell viability increased gradually following the increased concentration of tetramethylpyrazine. When PC12 cells were treated with 0.1 mM/L tetramethylpyrazine, the cell viability was increased significantly compared with the control group (*P* < 0.05). The TUNEL assay and flow cytometry also showed that tetramethylpyrazine could decrease the apoptosis induced by glutamate. In the result of RT-PCR, the transcriptional levels of Bcl-2, PKA, and CREB were increased and Bax was decreased. Meanwhile, Western blot showed that expression levels of Bcl-2, PKA, CREB, and p-CREB were increased and Bax was decreased.

**Conclusions:**

This study provided evidence that tetramethylpyrazine can protect against apoptosis caused by neuroexcitotoxicity and the protective mechanism is closely related to the activation of the PKA/CREB signaling pathway.

## 1. Introduction

The concept of excitotoxicity has come up to describe the excessive glutamate that acts on an excitatory receptor and causes cell death in 1969 [1, 2]. With the subsequent development, now, excitotoxicity is the pathological process in which neurons are damaged and killed because of excessive stimulation by neurotransmitters, such as glutamate and other similar substances [3, 4]. Hyperstimulation of neurotransmitters trigger a critical cascade of events which cause the aberration by energy deficiency, oxidative stress, mitochondrial dysfunction, or calcium overload, which leads to the abnormal intracellular pathways and cell-to-cell interactions [4–6]. This is a core mechanism in nervous system disease such as stroke, epilepsy, Alzheimer disease, Parkinson's disease, and Huntington disease [7–13]. Thus, multiple drugs have been invented and used to interrupt, influence, or temporarily halt glutamate-induced excitatory cascade and neuronal injury [14].

**Figure 1:**
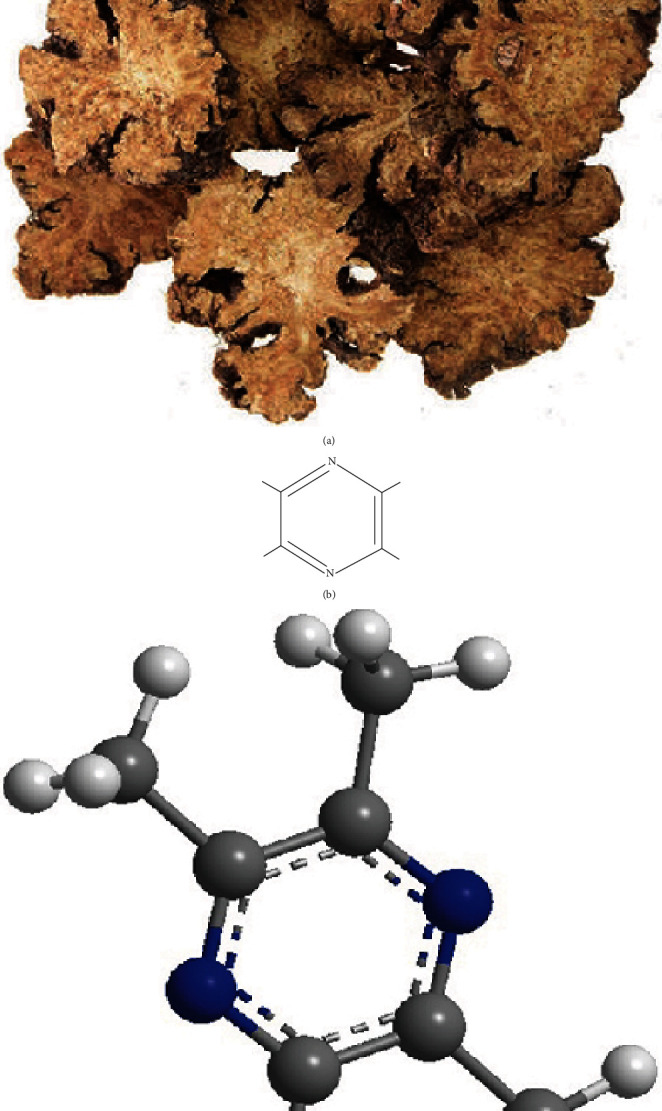
The herb of Ligusticum wallichii and 2D and 3D structure of tetramethylpyrazine: (a) the herb of Ligusticum wallichii which is one source of tetramethylpyrazine [36]; (b) 2D structure of tetramethylpyrazine; (c) 3D structure of tetramethylpyrazine.

Tetramethylpyrazine (TMP) is one main biologically active ingredient from *Ligusticum chuanxiong* (LC) (Figure 1(a)), one of the effective traditional Chinese medicines, belonging to the Umbelliferae family used for the treatment of neurovascular and cardiovascular diseases [15]. Its chemical name was also named 2,3,5,6-tetramethylpyrazine hydrochloride (Figures 1(b) and 1(c)), which has been used in the treatment of neurovascular diseases in China for several years [16–18]. According to the recent research, tetramethylpyrazine can achieve its therapeutic effect by antiapoptosis in a variety of diseases [19–21].

It has been reported that the PKA/CREB signaling pathway plays an essential role in learning and memory [22]. Protein kinase A (PKA) can phosphorylate and activate cAMP response element-binding protein (CREB) at its transcription activating site, one of phosphorylation-dependent nuclear transcription factors which can regulate the expression of neuronal survival and functional genes [23–25]. Recently, several studies have demonstrated that the PKA/CREB signaling pathway is essential for the process of excitotoxicity in neural disease, such as stroke, AD, and Parkinson's disease [26–28]. Several recent studies found that toxic substance caused the decrease in Bcl-2, as well as the increase in Bax which results in the apoptosis [29–32], while the activation of the PKA/CREB signaling pathway would show its cell-protective effect and inhibition of apoptosis through maintaining the normal level of Bcl-2 and Bax [33–35]. In this study, we wanted to focus on the protective mechanism of tetramethylpyrazine against neuroexcitotoxicity through the PKA/CREB signaling pathway.

## 2. Materials and Methods

### 2.1. Molecular Docking Study

We wanted to evaluate if PKA/CREB takes part in the protective effect of tetramethylpyrazine to explore their binding modes. The structure of tetramethylpyrazine (ID: 14296) was obtained from PubChem (https://pubchem.ncbi.nlm.nih.gov/). Meanwhile, the structure data of PKA (ID: 4zp3) and CREB (ID: 6E99) were obtained from the PDB database (http://www.rcsb.org/). We used Schrodinger software (version 9.2) to pretreat compound structures and generate multiple conformations. Protein structures were processed by removing hydrone and adding hydrogen atoms, and the Sitmap module was used to explore and define binding sites. Next, the liganddock module was used to simulate the molecular docking of compounds and proteins, and the docking score was evaluated by the docking score function. Simultaneously, the visual analysis module was used to observe the binding orientation of molecules and their targets.

### 2.2. Selection of Treatment Time and Concentration for the Glutamate Group as the Model Group

PC12 cells were inoculated into 96-well plates at the logarithmic phase. The different concentrations of glutamate (0, 0.05, 0.1, 0.2, 0.5, 1, 2, 5, 7, 10, and 12 mM/L) were added into the wells, when PC12 cells grew to 70%-80% of the culture wells. Each group was set with 5 multiple wells, and we detected them in different treatment times (3, 12, and 24 hours). After the above treatment, the medium containing the drug was sucked out, and 10 *μ*L CCK-8 reagent was added to each well and incubated at 37°C for 3 hours. The absorbability of each well was recorded by the microplate reader (*λ* = 450 nm); then, the cell viability was calculated.

### 2.3. The Influence of Cell Viability from Tetramethylpyrazine for the Excitotoxicity Model by Glutamate

The blank control group and glutamate groups (five groups) were set up, and each group was set with 5 multiple wells. After treatment of 10 mM/L glutamate for 12 hours, we sucked out culture medium and added different concentrations of tetramethylpyrazine (0, 0.02, 0.05, 0.075, and 0.1 mM/L) in the glutamate group, respectively, dealing with 2, 4, and 8 hours. Then, the medium was sucked out, and 10 *μ*L CCK-8 reagent was added to each well and then incubated at 37°C for 3 hours. The absorbability of each well was recorded by the microplate reader (*λ* = 450 nm).

### 2.4. Detection of Apoptosis Using the TUNEL Assay

The TUNEL assay was done using the TUNEL assay kit obtained from Abcam (USA). 4% paraformaldehyde was used to fix cells for 10 minutes; then, rinse fixation cells with DPBS. Each sample was covered with 100 *μ*L protein K (2 *μ*g/mL) and incubated at room temperature for 20 minutes. Soak with PBS (pH 7.4) for 5 minutes/time, 3 times. 50 *μ*L TUNEL test solution covered the cells, and incubate them at 37°C away from light for 1 hour. Soak with PBS (pH 7.4) for 5 minutes/time, 3 times. Then, 100 *μ*L DAPI (5 *μ*g/mL) working solution added to the cell reacts at room temperature for 5 minutes. PBS soaking was performed for 5 minutes/time, 3 times. Discard excess PBS and glycerol PBS sealing agent. The cells were then observed under the OLYMPUS BX60 microscope.

### 2.5. Detection of Apoptosis Using Flow Cytometry

Here, we divided the cells into three groups, namely, blank control group, glutamate group, and glutamate+tetramethylpyrazine group (0.1 mM/L, treatment for 8 hours). After digestion by pancreatic enzyme, these cells of the three groups were used by 4°C precooling PBS to rinse cells 2 times, and adjust the cell concentration to 1 × 10^6^/mL. After being resuspended and centrifuged twice in 1.5 mL centrifuge tubes with PBS, 200 *μ*L PBS was added into tubes. Each tube was added with 5 *μ*L FITC+5 *μ*L PI, incubated at room temperature in the dark environment for 10 minutes, and the apoptosis rate was tested on flow cytometry instrument.

### 2.6. RT-PCR for the Expression Level of Bcl-2, Bax, PKA, and CREB

PC12 cells were inoculated on a 6-well plate and divided into the blank control group (routine culture), glutamate group, and glutamate+tetramethylpyrazine group (0.1 mM/L, treatment for 8 hours). After digestion by trypsin and collection of cells, the total RNA was extracted using a RNA extraction kit (DP451, QIAGEN, Germany). The concentration of 1 *μ*L RNA sample was measured and stored at -80°C. The required RNA volume of each sample was calculated according to the reaction system of reverse transcription kit, and the corresponding cDNA sample was obtained by reverse transcription, which was stored at -80°C. The RT-PCR kit (AB-1454, Thermo, USA) was used to prepare the reaction system, and the corresponding gene expression was obtained by reaction in the fluorescence quantitative PCR instrument.

### 2.7. Western Blot Analysis

Western blot was used to detect the quantity of Bcl-2 (Ab194583, Abcam, UK), Bax (Ab32503, Abcam, UK), PKA (BS-0520R, Bioss, China), CREB (Ab32515, Abcam, UK), and p-CREB (Ab32096, Abcam, UK). PC12 cells were inoculated in the 6-well plate and intervened when the cell density reached 70%-80%. The PC12 cells were divided into the blank control group (routine culture), glutamate group, and glutamate+tetramethylpyrazine group (0.1 mM/L, treatment for 8 hours). After treatment, the medium was sucked out, each well was washed twice with precooled PBS, and 100 *μ*L lysate was added to treat cells for 20 minutes. The lysate was collected with a cell scraper and aspirated into a 1.5 mL EP tube. The lysate was centrifuged at 12000 r/minutes for 15 minutes, and the supernatant was collected and quantified by the BCA method. SDS-PAGE gel was prepared, after the separation gel was taken out and put into the electrophoresis solution. The PVDF membrane was activated in methanol solution for 10 minutes and then equilibrated in electrophoresis solution. The adhesive and membrane were carefully bonded together and put into the membrane transfer tank for membrane transfer. After the membrane was transferred, it was sealed in 5% skimmed milk powder for 1 hour and incubated overnight at 4°C. The next day, TBST was used to wash the PVDF membrane 3-5 times for 5 minutes each time. The second antibody was added and incubated at room temperature for 1 hour. Then, the PVDF membrane was washed by TBST 3-5 times; add the substrate developer and take color photos in the dark room.

### 2.8. Statistical Analysis

The data was shown as mean ± SD. Statistical comparisons were made using Student's *t*-test and one-way analysis of variance (ANOVA) by IBM SPSS Statistical software package (version 20). *P* < 0.05 was considered statistically significant.

## 3. Result

### 3.1. Molecular Docking Result

**Figure 2:**
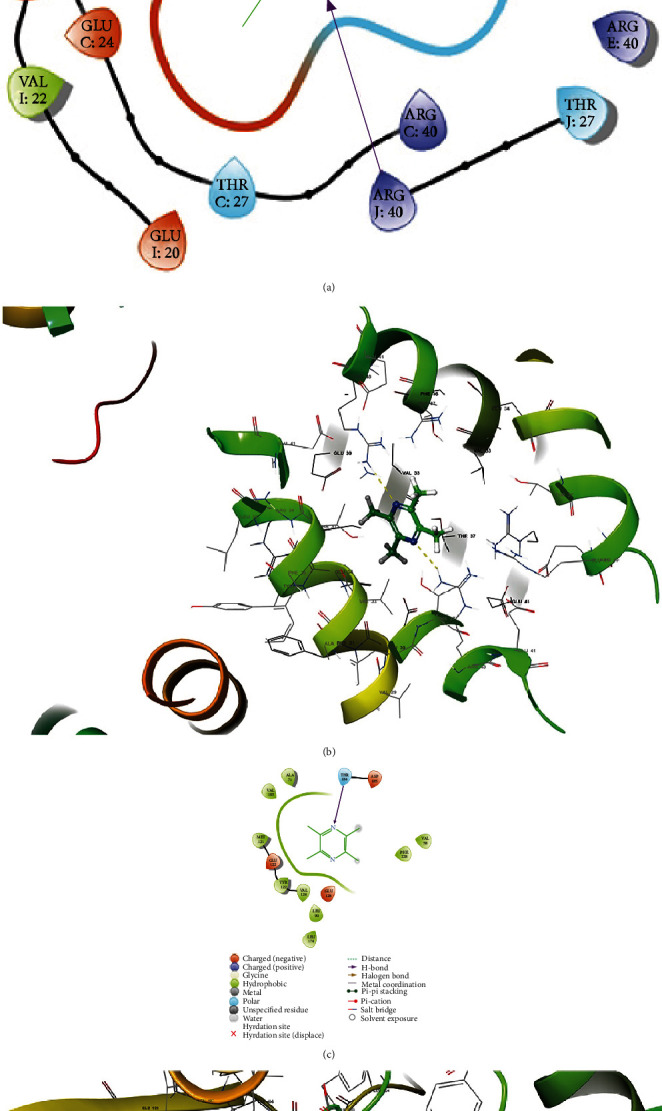
The molecular docking between tetramethylpyrazine and PKA/CREB pathway: (a). the 2D molecular docking between tetramethylpyrazine and PKA; (b) the 3D molecular docking between tetramethylpyrazine and PKA; (c) the 2D molecular docking between tetramethylpyrazine and CREB; (d) the 3D molecular docking between tetramethylpyrazine and CREB.

In general, in the ligand combined with its receptor, their bonding strength and mode would influence the activity of the receptor and subsequent signal transduction [37]. Therefore, we investigated the interaction and binding pattern between tetramethylpyrazine and the protein in the PKA/CREB pathway. It showed that tetramethylpyrazine had a good docking score (-5.312) with PKA which could form two hydrogen bonds with two PKA molecules in their arginine 40 (ARG 40) (Figures 2(a) and 2(b)). Meanwhile, there was moderate activity between tetramethylpyrazine and CREB in which the docking score was -3.838, and they could form one hydrogen bond in threonine 184 (THR 184) (Figures 2(c) and 2(d)).

### 3.2. The Different Effect for PC12 Cells Treated with Glutamate by Different Treatment Times and Concentrations

**Figure 3:**
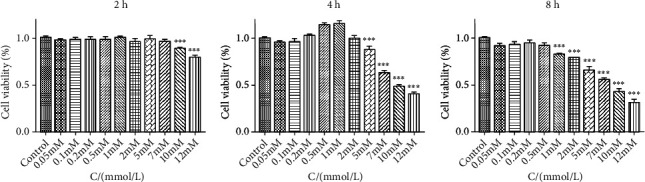
The different treat-effect for PC12 cells with different treatment times and concentrations of glutamate (∗∗∗ means *P* < 0.001 compared with control).

Compared with the control group, it showed that the activity of PC12 cells decreased gradually with the increase in the concentration and time of glutamate when the concentration of glutamate ranged from 5 mM/L to 12 mM/L. When PC12 cells were treated with 10 mM/L glutamate for 12 hours, the activity of the PC12 cell line (mean = 48.20%) was slightly lower than 50% compared with the blank control group (*P* < 0.05), which was close to half of the inhibitory concentration (IC50) and was used as the subsequent molding method (Figure 3).

### 3.3. The Influence of Cell Viability from Different Treatment Times and Concentrations of Tetramethylpyrazine against the Excitotoxicity Model by Glutamate

**Figure 4:**
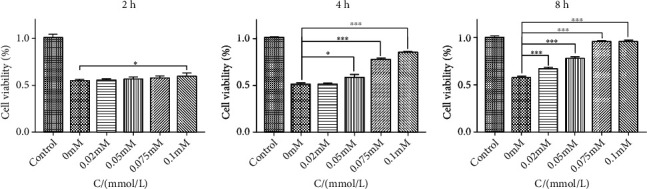
The influence of cell viability from different treatment times and concentrations of tetramethylpyrazine against the excitotoxicity induced by glutamate (compared with 0 mM/L tetramethylpyrazine; ∗ means *P* < 0.05 and ∗∗∗ means *P* < 0.001).

With the pretreatment of 10 mM/L glutamate for 12 hours, PC12 was treated with different concentrations of tetramethylpyrazine (0.02 mM/L, 0.05 mM/L, 0.075 mM/L, and 0.1 mM/L) for 2, 4, and 8 hours, respectively. When PC12 was treated with tetramethylpyrazine for 2 hours, only 0.1 mM/L tetramethylpyrazine showed the protective effect. And all concentrations showed their good protective effect reflected by CCK-8 (Figure 4) when treated for 8 hours. After 8 hours of treatment with 0.1 mM/L tetramethylpyrazine, the cell activity increased significantly, compared with the 0 mM/L tetramethylpyrazine group (glutamate group) (*P* < 0.001).

### 3.4. The Antiapoptosis Effect of Tetramethylpyrazine against the Excitotoxicity Induced by Glutamate Detected by the TUNEL Assay

**Figure 5:**
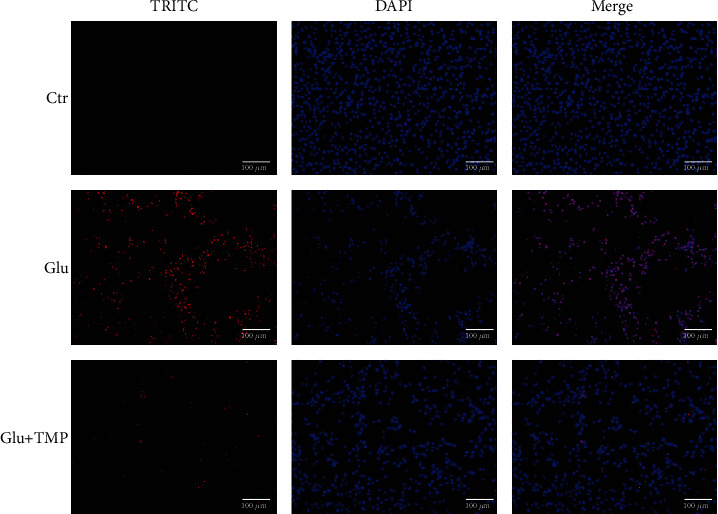
The protective effect of tetramethylpyrazine against apoptosis induced by glutamate detected by the TUNEL assay.

Compared with the control group, the apoptosis rate of the glutamate group was increased. Meanwhile, the tetramethylpyrazine group (with the pretreatment with 10 mM/L glutamate in the PC12 cell line for 12 hours, then the PC12 cell line was treated with 0.1 mM/L tetramethylpyrazine for 8 hours) could reduce apoptosis induced by glutamate compared with the glutamate group (Figure 5).

### 3.5. The Antiapoptosis Effect of Tetramethylpyrazine against the Excitotoxicity Induced by Glutamate Detected by Flow Cytometry

**Figure 6:**
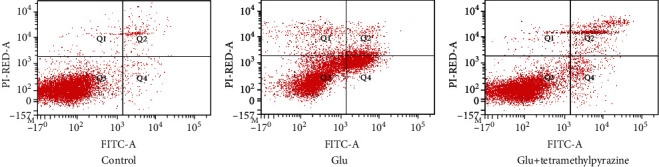
The protective effect of tetramethylpyrazine against apoptosis induced by glutamate detected by flow cytometry.

Compared with the control group, the apoptosis rate of the glutamate group was significantly increased, which indicated that the excitotoxicity of glutamate would increase apoptosis. Meanwhile, the tetramethylpyrazine group (with the pretreatment of 10 mM/L glutamate in the PC12 cell line for 12 hours, then the PC12 cell line was treated with 0.1 mM/L tetramethylpyrazine for 8 hours) could reduce apoptosis significantly compared with the glutamate group, which showed that tetramethylpyrazine had a protective effect against apoptosis induced by glutamate related to excitatory injury of nerve cells (Figure 6).

### 3.6. Tetramethylpyrazine Increased Bcl-2, PKA, and CREB and Reduced Bax Level

**Figure 7:**
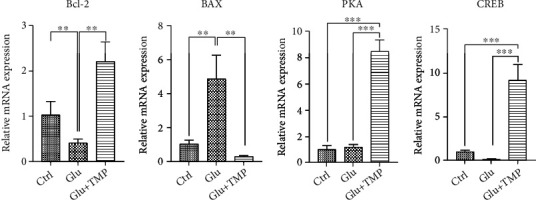
The RT-PCR for the transcription level of Bcl-2, Bax, and PKA in the control group, Glu group, and Glu+TMP group (∗∗ means 0.001 < *P* < 0.01; ∗∗∗ means *P* < 0.001).

We used RT-PCR to detect the transcriptional level of Bcl-2, Bax, PKA, and CREB. The transcription level of Bcl-2, PKA, and CREB increased significantly, while the transcription level of Bax decreased in the glutamate+tetramethylpyrazine group compared with the glutamate group (∗∗∗ means *P* < 0.001) (Figure 7).

**Figure 8:**
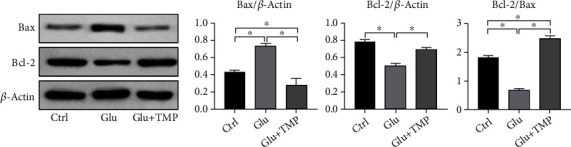
The expression level of Bax and Bcl-2 in the control group, Glu group, and Glu+TMP group (∗ means *P* < 0.05).

**Figure 9:**
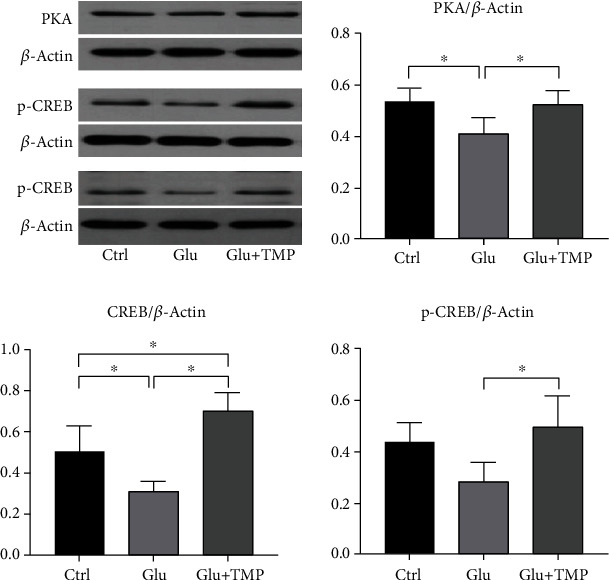
The expression level of PKA, CREB, and p-CREB in the control group, Glu group, and Glu+TMP group (∗ means *P* < 0.05).

The results of Western blot showed that the expression level of Bax protein in the glutamate group was significantly increased compared with that in the blank control group which was without any treatment, and the expression level of Bcl-2 protein was significantly decreased simultaneously. It showed that the excitotoxicity effect of neurons induced by glutamate could cause the increase in apoptosis (Figure 8). Compared with the glutamate group, the ratio of Bcl-2 to Bax and the levels of Bax, PKA, CREB, and p-CREB were increased, and the expression level of Bcl-2 was decreased in the tetramethylpyrazine group (with the pretreatment of 10 mM/L glutamate in the PC12 cell line for 12 hours, then the PC12 cell line was treated with 0.1 mM/L tetramethylpyrazine for 8 hours) (Figures 8 and 9). It showed that tetramethylpyrazine could effectively protect the neuroexcitotoxicity induced by glutamate.

## 4. Discussion

With no doubt, excitotoxicity plays a key role in the nervous system and psychiatric diseases, manifesting neural degeneration and/or neuronal death [38]. Meanwhile, one previous report has demonstrated the connection between the PKA/CREB signaling pathway and neuroprotection [27]. So, we first used molecular docking techniques to investigate the possible interaction of tetramethylpyrazine with PKA and CREB. Here, we used Schrodinger software to pretreat compound structures and generate multiple conformations. The results showed that tetramethylpyrazine had a good docking activity (docking score -5.312) with PKA forming two hydrogen bonds with two PKA molecules in their arginine 40 (ARG 40) (Figures 2(a) and 2(b)). The interaction between them may influence the downstream of the pathway momentously, including the activity of this pathway by autophosphorylation or catalytical phosphorylation possibly. Thus, tetramethylpyrazine likely regulates the PKA/CREB signaling pathway against excitotoxicity probably in neurological disorders because the latter was pivotal in neuroexcitotoxic diseases [26–28].

Then, the PC12 cell line was treated with different doses and time gradients of glutamate to build the model of neuroexcitotoxicity, because glutamate is one important element in governing physiological balance of CNS, and the excessive activation of glutamate receptors is neurotoxic [4, 39]. With the increase in glutamate concentration and action time, PC12 cells' activity decreased significantly, suggesting that the degree of apoptosis caused by excitotoxicity depends on the action time and dose of glutamate. This result was similar to that of previous studies that excessive activation of the glutamate receptor could lead to a number of harmful results [40], which may cause a number of neural diseases, such as stroke, epilepsy, and Alzheimer disease [41]. Here, we chose 10 mM/L glutamate treating PC12 cells for 12 hours as the dose and action time for the subsequent model of neurotoxicity. We chose different concentrations and action times of tetramethylpyrazine to explore its protective effect. Consequently, the protective effect was gradually enhanced with the increase in concentration and action time of tetramethylpyrazine. Thereafter, we detected the apoptosis of PC12 by flow cytometry, and the apoptosis rate of the glutamate group was significantly increased, while apoptosis was decreased after the addition of tetramethylpyrazine significantly. Then, we detected the transcription and expression level of Bcl-2 and Bax proteins. Compared with the glutamate group, we found that the transcriptional and expressional level of Bcl-2 increased and Bax decreased in the glutamate+tetramethylpyrazine group. It is well known that the ratio of Bcl-2 to Bax is a key factor in regulating cell apoptosis; the higher the ratio is, the more the cells resist apoptosis and are inclined to survive [42, 43]. Therefore, the increase in the ratio of Bcl-2/Bax suggests that tetramethylpyrazine has an obvious antiapoptosis effect caused by neuroexcitotoxicity induced by glutamate.

The PKA/CREB signaling pathway not only plays an important role in the physiology of the nervous system but also plays a key role in pathology in a variety of nervous system diseases induced by neuroexcitotoxicity. Recently, a study has shown that the PKA/CREB pathway took part in the mechanism of apoptosis [14]. The activation of the PKA/CREB signaling pathway can activate the transcriptional activity of Bcl-2, inhibit cell apoptosis, and promote postinjury repair, reflecting the connection between the PKA/CREB signaling pathway and neuroprotection [15, 27]. In our experiment, we found that the transcriptional level of PKA and CREB increased; meanwhile, the expression level of PKA, CREB, and p-CREB also increased correspondingly after the treatment with tetramethylpyrazine (Figures 7 and 9). All these suggested that tetramethylpyrazine could activate the PKA/CREB signaling pathway, subsequently promote the expression of antiapoptosis proteins (Bcl-2), and inhibit the expression of apoptosis proteins (Bax), ultimately inhibiting apoptosis. At present, tetramethylpyrazine as an injection has been used in stroke patients in China for several years, while it has not been utilized in nervous system diseases such as Alzheimer's disease and epilepsy which are also related to the neuroexcitotoxicity. Thus, it may be helpful in the treatment of neural system disease caused by neuroexcitotoxicity. Meanwhile, tetramethylpyrazine comes from the traditional Chinese medicine Ligusticum chuanxiong, which is widely used in head-related diseases, especially nervous system diseases. Therefore, in view of its potential neuroprotective effect, we need more effort to focus on whether tetramethylpyrazine can be utilized in the treatment of other nervous system diseases further.

## 5. Conclusion

In conclusion, tetramethylpyrazine can protect against the neuroexcitotoxicity which plays an import role in nervous system disease induced by glutamate, and the protective mechanism is closely related to the activation of the PKA/CREB signaling pathway.

## Data Availability

The data used to support the findings of this study are available from the corresponding author upon request.

## Abbreviations

LC: Ligusticum chuanxiong
TMP: Tetramethylpyrazine
Glu: Glutamate
PKA: Protein kinase A
CREB: cAMP response element-binding protein
CNS: Central nervous system.
